# Joining the dots: maternal cytomegalovirus, HIV and the placenta

**DOI:** 10.1098/rstb.2024.0400

**Published:** 2025-11-06

**Authors:** Brandon Paarwater, Doty Ojwach, Nicole Prins, Rana Chakraborty, Erica Johnson, Viviane Schuch, Mark R. Schleiss, Clive M. Gray, Helen Payne

**Affiliations:** ^1^ Reproductive Immunology Research Consortium in Africa, Biomedical Research Institute, Stellenbosch University Faculty of Medicine and Health Sciences, Cape Town, Western Cape 7505, South Africa; ^2^ Pediatric Infectious Diseases, University of Miami Miller School of Medicine, Miami, FL 33146, USA; ^3^ Department of Microbiology, Biochemistry and Immunology, Morehouse School of Medicine, Atlanta, GA 30310, USA; ^4^ Division of Paediatric Infectious Diseases, University of Minnesota Medical School, Minneapolis, MN 55454, USA; ^5^ Paediatric Infectious Disease, Imperial College London, London SW7 2AZ, UK

**Keywords:** cytomegalovirus, HIV, CMV–HIV coinfection, placenta, infant ontogeny, immunity, immune dysregulation, transmission

## Abstract

Elucidating how HIV and cytomegalovirus (CMV) co-infection in pregnancy disrupts immune homeostasis at the maternal–fetal interface can provide a foundation for developing intervention strategies to improve infant and maternal health. Of importance is the identification of infant immune dysregulation, especially in HIV-exposed uninfected newborns (HEU). Although vertical transmission of HIV has been substantially mitigated by antiretroviral treatment, the incidence of adverse birth outcomes remains significantly higher in these infants than in those born to mothers without HIV. We hypothesize that the immune status of mothers during pregnancy impacts upon the placenta, in turn affecting infant immunity. We explore this hypothesis by examining data from key studies and clinical cohorts, aiming to ‘join the dots’ to form a body of knowledge to catalyse development of more comprehensive studies. Our narrative represents a thought piece incorporating events around HIV and CMV during the antenatal period, the impact of maternal immunity and immune events in the placenta, and how these factors produce a ‘knock-on’ effect on infant immune ontogeny and susceptibility to poor health. We introduce the concept of vertical transmission of inflammation, where in the absence of viral transmission, the effects of a maternal inflammatory milieu could profoundly impact birth and infant outcomes via placental dysfunction.

This article is part of the discussion meeting issue ‘The indirect effects of cytomegalovirus infection: mechanisms and consequences’.

## 1. Introduction

Human cytomegalovirus (CMV), a β-herpesvirus, is the most common congenital infection worldwide, affecting 0.67% of births [1], resulting in severe neurodevelopmental delay and hearing impairment in 25% of affected infants [2]. CMV global seroprevalence is approximately 80% and reaches almost 100% in low- and middle-income countries, albeit with substantial geographical variation [3,4]. Although many seropositive individuals remain asymptomatic throughout life, CMV is a chronic latent infection with intermittent reactivation, such as during pregnancy, particularly for mothers living with HIV [5–7]. Within the complex landscape of maternal–fetal health, HIV and CMV co-infection introduces a confluence of challenges during and after pregnancy. The propensity of HIV to undermine host immunity magnifies the impact of CMV. However, the tandem effects of ongoing HIV and CMV co-infection in pregnancy are poorly characterized. There is therefore an increased need to understand the immunological landscape of CMV and develop effective and safe therapeutic interventions to reduce the burden of fetal CMV disease and the indirect effects that CMV reactivation in pregnancy can cause.

Elucidating mechanisms that disrupt immune homeostasis at the maternal–fetal interface can provide valuable insights into the interplay between HIV and CMV coinfection that contributes to adverse clinical outcomes reflected by aberrant infant immunity. Vertical transmission of HIV has been mitigated by the successful implementation of antiretroviral treatment (ART) prior to, during and after pregnancy [8]. Despite these successes, the incidence of adverse birth outcomes and co-morbidities in newborn HEUs remains significantly higher than in infants born to mothers who do not have HIV. It is hypothesized that viral-associated inflammation in women living with HIV and CMV co-infection during gestation impacts placental health, which in turn has negative effects on neonatal and infant immunity and renders these children more susceptible to co-morbidities. Viral-induced inflammation forms the basis of this opinion piece, where we examine data from several cohorts to understand the impact of single and multiple maternal infections on placental immunity and how these impact upon immune ontogeny in postnatal life.

We will attempt to *join the dots* linking HIV, CMV and placental immunology to infant immunity and early life health outcomes. This opinion piece will outline events in the placenta during maternal co-infection and propose mechanisms by which HIV and CMV can disrupt immune homeostasis linking mother and infant, highlighting key studies that support or refute this narrative.

## 2. Dots

### (a) Dot 1: Antenatal cytomegalovirus infection and immunity

Primary CMV infection is when a CMV-seronegative individual acquires the virus for the first time. This can occur in 1–3% of pregnant women, with an overall vertical transmission rate to the fetus of approximately 40% [9–11]. When primary CMV infection occurs during the first trimester of pregnancy, a transmission rate to the fetus of 24% has been reported, of whom 38.4% develop severe central nervous system impairment. Although transmission is more common in the second trimester (38%), rising to 72% in the third trimester, severe sequelae are only seen in 3.4% and 0% of infants infected in these trimesters, respectively [9–11]. This is unsurprising and probably reflects disruption of embryogenesis during the first trimester, coupled with a lack of immune protection by the fetus. Understanding why only a proportion of fetuses are so severely affected is critical in developing interventions to prevent, treat and manage congenital CMV (cCMV). The fact that 76% of pregnant women with primary CMV infection do not transmit the virus, and that 61.6% of infants with cCMV exhibit no severe sequelae [9–11], suggests the existence of some form of protective pre-existing maternal immunity—including within the placenta—that can prevent transmission and subsequent disease. Understanding how maternal immunity can prevent transmission and mitigate severe adverse infant outcomes warrants further investigation, and such studies may identify key protective immune modulators.

In the CMV seropositive host, CMV remains latent, typically in monocytes, and it can be detrimental during pregnancy if reactivation occurs, which can be triggered through pathways activated by inflammation, infection and injury [12–14]. Alternatively, the host can be re-infected with a different strain of CMV [15]. These means of transmission are known as non-primary CMV infection. CMV infection is recognized to occur in 0.1–0.5% of pregnancies via reactivation or re-infection with a different strain of CMV, with a vertical transmission rate of 1–6% to the fetus [16,17]. Therefore, pre-conceptual immunity can reduce transmission but does not completely abrogate the risk. Some investigators have reported partial protection against intrauterine transmission as being associated with the presence of CMV-specific adaptive immune responses [18]. Others have documented cross-reactive immunity in CMV-seropositive individuals after activation of tissue-resident memory CD8⁺ and CD4⁺ T cells in the decidua during reinfection [19]. Whether transmission is primary or non-primary does not mitigate fetal damage, since there is no difference between cCMV disease severity in infants infected between the two means of transmission [20].

CMV exhibits broad cell tropism, inducing productive infection in cells of epithelial, fibroblast and endothelial lineage [21,22]. During CMV infection or reactivation, CD8+ T cells are thought to be key mediators of protective immunity, and successful viral antigenic clearance is achieved through priming of naive CD8+ T cells, via T-cell receptor (TCR)-peptide-major histocompatibility complex restriction, after which cells clonally expand and differentiate into different CMV-specific memory CD8+ T subsets. These antigen-experienced T cells remain present in the systemic immune system after primary infection, including residence in tissues such as bone marrow, lymph nodes, lung and spleen [23]. CMV-specific T cells can make up more than 10% of the total CD8+ T-cell pool in seropositive individuals [24,25] and consist of a mix of effector memory (EM) and terminally differentiated EM CD45RA (TEMRA) T cells [26,27]. CMV-specific TEMRA cells are understood to be more immune-senescent [28], and so CMV antigen pp65 on the expanded T-cell memory pool probably reflects age-related changes [29,30]. Furthermore, these cells may represent inflationary CMV-specific memory T cells [31], which are long-lived CD8+ T cells that gradually increase over time in response to persistent antigen stimulation, probably in conjunction with low levels of viral reactivation [32]. Conversely, non-inflationary T cells also undergo expansion but contract and form stable, low-frequency memory pools during CMV infection [32].

Expanded inflationary T-cell populations have been well described in the context of CMV infection [33] and were initially described in response to continuous accumulations of viral antigen-specific CD8+ T cells to viruses that establish latency [31–33]. How these possible inflationary CMV-specific CD8+ T cells contribute to protection or pathology is open to question. What is unknown is whether these cells can expand during pregnancy and can mitigate *in utero* transmission of CMV. If there is memory inflation during pregnancy, can these cells migrate into the placenta? If they are found in the placenta, is this an origin of villitis of unknown etiology or can they provide some level of protection?

What is also not known is the impact of CMV and HIV coinfection. Do CMV-specific CD8+ T cells exhibit a more senescent (exhausted) phenotype in pregnant women living with HIV? And could this contribute to increased susceptibility or altered immune responses in infants with congenital CMV? [34].

### (b) Dot 2: Cytomegalovirus and the placenta

In cases of confirmed cCMV infection, typical placental histopathology can include chronic villitis, plasma cell infiltration, viral inclusion bodies and hemosiderin deposits [35]. Interestingly, in one study of 27 mother–infant dyads with confirmed cCMV, CMV infection was identified in only 44 and 70% of placental tissue, respectively, using immunohistochemistry and PCR [35], which may reflect capacity for cellular recovery and immune control within the placenta.

Placental trophoblasts are at the core of a successful pregnancy, acting as a structural and bio-molecular barrier between the mother and the fetus. Additionally, these cells play a critical role in educating immune cells and shaping the immunological profile at the maternal–fetal interface [36]. For successful transmission of CMV to the fetus, the virus first needs to traverse placental barriers comprising syncytiotrophoblast layers and the decidua–trophoblast interface [37,38], and in fact, CMV has been seen to suppress trophoblast syncytialization, thereby facilitating transmission [39]. CMV is known to circumvent host immunity by downregulating human leucocytic antitgen-C (HLA-C) allele expression, which hinders trophoblast invasion into the maternal uterine tissue during early pregnancy. Downregulated HLA-C reduces both T-cell recognition [38,40] and, T-cell activation [41], and it may affect vascular remodelling by natural killer (NK) cells, potentially causing placental insufficiency [38]. *In vitro* studies have shown that first-trimester syncytiotrophoblasts resist CMV infection, while cytotrophoblasts (CTBs) remain susceptible, impacting differentiation and invasion [36,38]. Similarly, extravillous trophoblasts maintain HLA-G despite CMV infection, possibly maintaining immune tolerance while impairing HLA-C [42]. CMV infection of extravillous trophoblasts (HTR-8/SVneo) triggers inflammation-induced cell death, which may impair spiral artery remodelling at the maternal–fetal interface. Collectively, these immune responses to CMV within the placenta probably reduce blood flow, cause local hypoxia and contribute to adverse birth outcomes [43] ranging from miscarriage to prematurity and intrauterine growth restriction.

### (c) Dot 3: Cytomegalovirus, HIV and the placenta

As discussed above, CMV is ubiquitous among pregnant women in sub-Saharan Africa [44,45] and it may amplify HIV transmission risk by facilitating HIV replication in placental cells [46]. And *vice versa*—CMV reactivation more commonly occurs during HIV coinfection, even when ART is effectively suppressing HIV replication. Rates of cCMV are up to fivefold higher in HIV seropositive mothers compared with HIV seronegative mothers [47]. Nonetheless, maternal CMV–HIV coinfection remains underexplored [37]. Johnson *et al.* [46] demonstrated that CMV infection enhances the susceptibility of placental macrophages (Hofbauer cells) and trophoblasts to HIV-1 by upregulating HIV-1 coreceptors such as CCR5 and increasing proinflammatory cytokines that promote viral replication. Additionally, CMV-induced immune activation and disruption of placental barrier integrity may create a more permissive environment for HIV-1 entry, replication and potentially transmission to the fetus. Additionally, CMV can increase HIV transmission risk by promoting immune activation, increasing viral load, causing damage to mucosal barriers and interacting with HIV in ways that further compromise the immune system. CMV DNAemia linked to vertical transmission persists even with low antenatal HIV RNA loads [48].

Placental trophoblasts tightly regulate immune sensing interactions in the placenta, and chronic maternal infections such as HIV and CMV can combine to disturb immune tolerance, which in turn may precipitate HIV or CMV transmission to the fetus [49–52]. Trophoblasts play a crucial role in detecting pathogen-associated molecular patterns from bacteria, viruses (including HIV and CMV) [53], fungi and parasites through their expression of pattern recognition receptors, notably Toll-like receptors (TLRs) and Nod-like receptors (NLRs) [54–56]. TLRs are categorized into surface (TLR 1, 2, 4 and 5) or endosomal (TLR 3, 7, 8 and 9) receptors, with their presence detected in both early and term human placentas [57]. Notably, TLR expression by trophoblasts influences the recruitment, differentiation and regulation of immune cells across the different phases of gestation [57,58]. An *in vitro* study examining trophoblast cell lines cultured with all 10 TLRs demonstrated downstream signalling pathways that elicited diverse TLR-mediated immune activation ranging from a regulated protective response promoting pregnancy maintenance to exaggerated pathological responses with potential detrimental effects on the pregnancy [59].

Host immune responses to infection and ensuing placental inflammation can impact fetal outcomes, as seen in mice [60] and human models [39,61]. Vertical transmission of CMV hinges on gestational timing. Epidemiological studies demonstrate that while cCMV disease is largely confined to first trimester infection, CMV transmission rates increase as pregnancy progresses. However, *in vitro* studies have demonstrated that first-trimester villous trophoblasts may be more susceptible to infectious replication after infection with laboratory strain AD169 CMV relative to those in the third trimester [62]. This might reflect the difference between the fetal immune response in the first compared with the third trimester. How CMV–HIV coinfection affects placental micro-anatomy and immune networks is not known and may be key to understanding transmission risks, as well as neonatal and infant outcomes [52,63].

**Figure 1.**
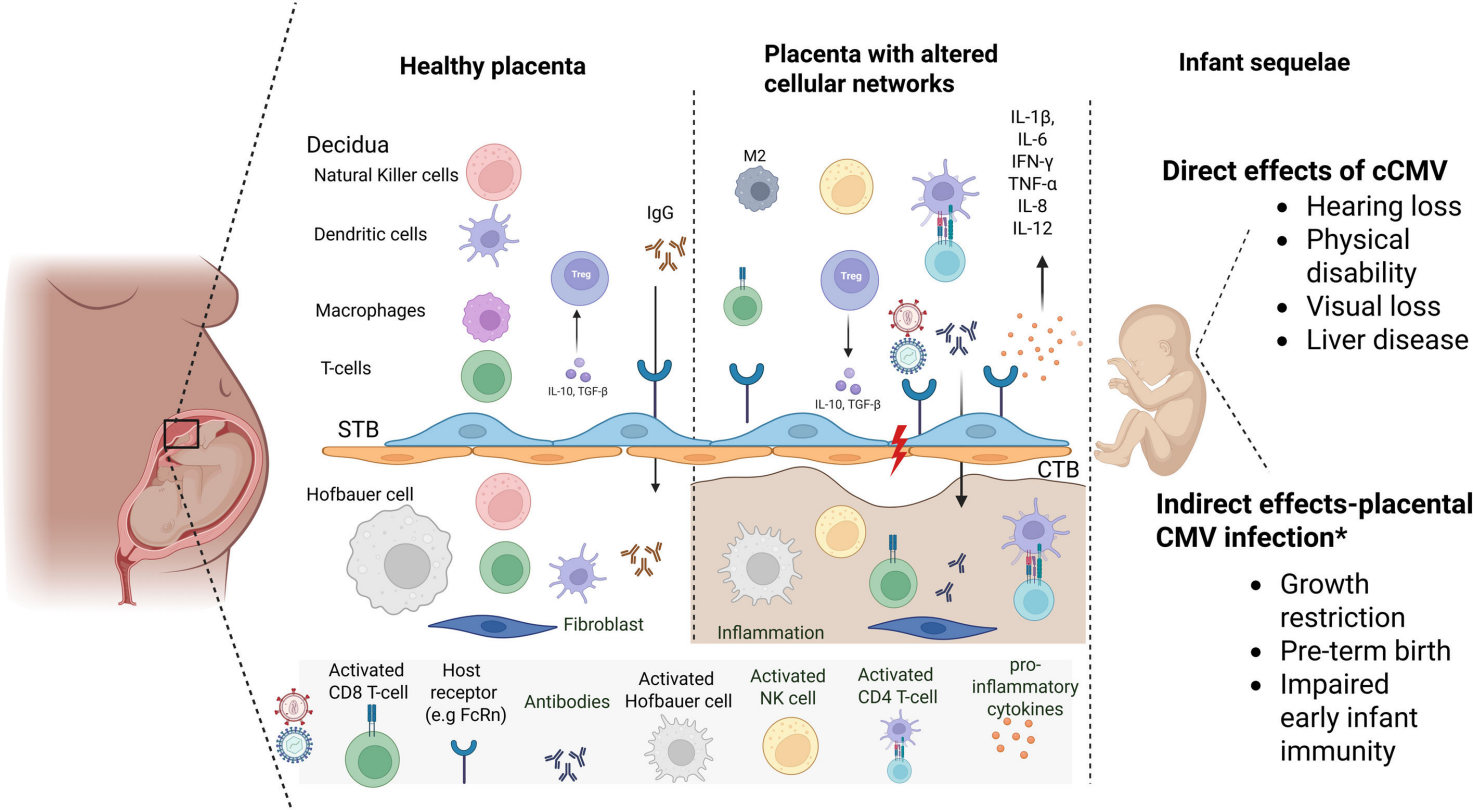
Maternal inflammation transmission to placental cellular networks has direct and indirect consequences for infant health sequelae. In a healthy placenta, decidual immune cells are in homeostatic balance associated with a physiologically successful pregnancy. In the placenta exposed to HIV alone, or with CMV co-infection, the effective barrier of cytotrophoblasts (CTBs) is breached owing to local inflammation with attendant elaboration of inflammatory cytokines and subsequent tissue damage. Viral entry through syncytiotrophoblasts (STBs) can potentially occur. A breach of this barrier provides the opportunity for pathogen transmission from maternal blood into placental villi, with a risk of direct and indirect effects of cCMV infection. In the absence of viral transmission, there is a pro-inflammatory immune response in the placenta causing aberrations in both genes regulating cellular immunity, as well as genes related to a healthy functioning *in utero* state. *Refers to the indirect effect of CMV exposure to the placenta and not directly to the fetus. Image created using biorender.com.

An extension of the phenomenon of vertical transmission of CMV is the concept of *vertical transmission of inflammation* (or *inflammation transmission*), which can perturb placental immune networks in the absence of viral transmission, as demonstrated in maternal SARS-CoV-2 infection [64]. The concept of inflammation transmission is illustrated in figure 1. During pregnancy, there is transient suppression of T-cell-mediated immunity that enables tolerance of the semi-allogeneic fetus, which indirectly increases susceptibility to infections, including HIV and CMV^4^ [65]. Infiltration by inflammatory lymphocytes, plasma cells and/or macrophage cells, and concomitant release of pro-inflammatory mediators, can cause pathologic alterations that can impair maternal–fetal vascular exchanges, resulting in small-for-gestational age infants and prematurity [66].

Maternal HIV is known to result in persistent immune activation despite HIV suppression with ART [67,68], driving inflammation-mediated interference with placental function [52,69]. Administration of antepartum ART may reduce the impact of maternal HIV on pregnancy outcomes, reducing viral load and associated inflammation [70]. However, despite increased ART coverage, *in utero* HIV/ART exposure is associated with low birthweight in infants, preterm labour and stillbirth [52,70,71]. These adverse outcomes may be attributable to the possible ‘off-target’ effects of maternal ART during gestation, which can impact placental vascularization; this possibly causes fetal growth restriction by modulating progesterone [72,73], which in turn impacts decidualization and modifyies spiral artery remodelling [74]. Collectively, persistent placental immune activation may, in large part, be driven by the maternal immune environment and not solely by viral transmission. Such inflammation transmission could probably be the result of HIV and CMV co-infection during pregnancy, which results in placental inflammatory-mediated dysfunction giving rise to adverse birth outcomes.

### (d) Dot 4: Placental immune cells reflect maternal immunity

We have previously reported that placentae from women with HIV exhibit altered CD4 : CD8 ratios in the decidua membranes and elevated tissue-resident CD8+ T cells in villous tissue [66]. The presence of these CD8+ T cells, concentrated in and around fetal capillaries, correlated with maternal HIV viraemia prior to provision of ART, initiated at the start of the third trimester [67]. A large proportion of these CD8+ T cells in the fetal villous tissue were of a late to terminal memory differentiated state in placentae collected at term from women living with HIV. This would suggest that T cells were either engaging with antigen *in utero* or were virtually primed within the inflammatory environment of the placenta [66,75–77]. These changes may contribute to an altered infant immunophenotype at birth, as has been seen in HEU infants with a high proportion of differentiated CD4+ and CD8+ T cells [78], and elevated pro-inflammatory cytokines [79]. Together, these findings indicate that maternal HIV infection shapes placental immune profiles in the absence of HIV transmission and that it potentially promotes immune activation over tolerance to the fetus [70].

### (e) Dot 5: CD8^+^ cytomegalovirus-specific T cells in the placenta

The maternal–fetal interface is made up of the decidua—a maternally derived tissue—and extravillous trophoblasts, which are fetally derived. Tissue-type specific immune responses have been well characterized for the decidua parietalis and decidua basalis, but not necessarily for the villous tissue. To understand transplacental viral transmission from the systemic to the localized placental microenvironment, examining events in the villous tissue is important. As discussed in Dot 1 and Dot 2, immune control of viral infection is dependent on robust CD8^+^ T cells [80]; however, whether CMV-specific CD8+ T cells can (a) be found in the villous tissue and (b) cause pathology are important questions that should be explored.

If CMV-specific CD8+ do migrate from the maternal circulation into the placenta, the nature of such migration is ill-described. CMV-specific CD8^+^ T cells have been found in the decidual membranes during human pregnancy and were postulated to maintain immunity against placental infection [81]. The absence, or low expression of perforin and granzyme B [82,83] may bring into question whether these are indeed protective, considering they also expressed the exhaustion marker PD-1 [81]. Whether CD8+ T cells in the villous tissue, as described in Dot 4, are potentially protective or markers of viral acquisition remains an enigma.

### (f) Dot 6: Distinct immune responses to HIV and cytomegalovirus in Hofbauer cells across gestation highlight evolving placental immune dynamics

Placental macrophages (Hofbauer cells (HCs)) are thought to be key mediators involved in HIV and CMV transmission to the fetus [84,85]. HCs are a population of placental macrophages that contribute to various biological processes, including maintaining tissue homeostasis, sensing viral infections and mounting cell-autonomous antiviral immune responses.

These cells are characterized by a high level of plasticity, whereby their phenotype and function are strictly regulated by the local microenvironment. The placental milieu is dynamic throughout gestation, cycling between pro- and anti-inflammatory states during fertilization, implantation, maintenance, labour preparation and parturition. Thus, maintaining the balance of macrophage functional polarization (M1 pro-inflammatory and M2 anti-inflammatory) at the maternal–fetal interface is essential for a healthy pregnancy. Recent studies have demonstrated that HCs are activated and abundant in early pregnancy and possess molecular signatures specific to inflammatory phenotypes early in gestation compared with term [86]. In addition, HCs can recognize and respond to viral pathogens temporally at the various stages of pregnancy [86], which may influence postnatal outcomes.

In early gestation, we have demonstrated that CMV- and HIV-exposed HCs both exhibit extensive downregulation of genes involved in developmental and vascular pathways related to hypoxia, cellular differentiation (early and late oestrogen response), coagulation, UV response and Kras signaling [87]. Among the pathways consistently downregulated across both viruses and gestational stages, we identified apical junctions and epithelial–mesenchymal transition as potentially important variables for future study. Apical junctions are crucial for cell–cell adhesion and barrier function, and epithelial–mesenchymal transition (EMT) is essential for tissue remodelling and trophoblast invasion.

Notably, CMV exposure was uniquely associated with disruption of WNT/β-Catenin signalling, an essential regulator of trophoblast differentiation, embryonic development and early neuronal development. This finding is supported by reports demonstrating that CMV infection dysregulates the canonical WNT/β-catenin signalling pathway in extravillous trophoblasts [88] and impairs trophoblast progenitor cell differentiation [89], which may contribute to placental dysfunction and adverse fetal outcomes [90]. Regardless of the virus, early gestation HCs are proposed to exhibit a core antiviral response characterized by the upregulation of interferon-driven effectors incuding the following immune-regulated genes: GBP1, GBP2, GBP4, IRF1, TAP1, APOL6 and ETV7 [87]. This common signature reinforces the concept that HCs act as a frontline defence against viral pathogens, consistent with established models of cell-autonomous immunity. In addition to this shared response, HIV exposure distinctly modulates genes involved in chemokine signalling and antigen-processing activities (e.g. CXCL10 and LAMP3). These selective changes suggest that HIV exposure may reshape the immunological landscape of the placenta, potentially influencing fetal immune development and susceptibility to infection.

At term, CMV-exposed HCs upregulate interferon/antiviral pathways, with enrichment of interferon-α/γ responses, TNFα signalling via NF-κB and apoptosis. These changes indicate a potent antiviral response that, while protective, may also lead to tissue damage if unchecked. In parallel, both HIV- and CMV-exposed term HCs showed increased IL6/JAK/STAT3 signalling, reflecting a distinct yet overlapping pattern of immune activation [91]. Importantly, the upregulation of these inflammatory pathways, together with the activation of allograft rejection and apoptotic mechanisms in CMV, could predispose placental tissues to immune-mediated damage and compromise their ability to respond to co-infections [87]. Collectively, these findings advance our understanding of the evolving placental immune landscape during gestation and highlight how viral infections may compromise placental integrity, thereby contributing to adverse pregnancy outcomes and facilitating vertical transmission.

### (g) Dot 7: Immunity in early life and health outcomes

The impact of exposure to any viral infection *in utero* is crucially dependent upon the age of the fetus and corresponding phase of embryological and immune development. While maternal T-regulatory cell dysfunction probably contributes to the pathogenesis of pregnancy complications, the impact on infant immunity is not well described [92].

Fetal or maternal antigen-specific regulatory cells are essential for maintaining immune tolerance to the semi-allogeneic fetus during pregnancy [61,93]. T regulatory cells produce minimal levels of interferon-γ (IFN-γ) and interleukin-12 (IL-12) compared with activated T helper 1 and NK cells. They can suppress Th1 differentiation and may inhibit effective clearance of certain pathogens, such as viruses and intracellular bacteria, by dampening pro-inflammatory responses [94]. High levels of viral antigenic exposure *in utero* in the first trimester may render the fetus in a state of immune tolerance, especially if the antigens are presented during a critical window of immune development [90]. Exposure to antigens during this period, especially without co-stimulatory signals, may lead to clonal deletion, anergy and induction of fetal T regulatory cells [95]. While this process may have a protective effect upon the fetus through avoidance of damaging pro-inflammatory responses, direct tissue infection from CMV can cause lytic damage and significant neuropathology that are the hallmark of symptomatic cCMV disease [96].

The notion of downregulation of inflammatory pathways is supported by recent transcriptomic profiling of infants with cCMV [97]. This differed significantly from CMV-uninfected controls, although this pattern was seen in both symptomatic and asymptomatic infants, suggesting it may be a result of CMV infection and not necessarily CMV disease. Neonates with cCMV may exhibit reduced or absent immune responses to CMV stimulation [98], along with a profile of higher frequencies of terminally differentiated and exhausted T cells [34,99]. However, in most infants with cCMV, this appears to be a CMV-specific response, although data are limited. Immune tolerance towards CMV may persist during the neonatal period, although it is not known whether this has a compromising effect after the fetal–neonatal period, as children with cCMV do not usually have issues with recurrent or unusual infections.

Evidence exists showing that HEU infants face a higher risk of mortality, malnutrition, stunting and hospital admissions owing to infectious causes compared with their HIV-unexposed uninfected counterparts [100,101]. In part, the increased rate of infectious complications may be attributable to abnormal T-cell memory differentiation, preceded by lower TCR Vβ clonotypic diversity and possible TCR clonal deletion within naive T cells [102]. However, it is unclear what drives this reduced diversity. Is it the presence of HIV antigens, potentially alongside other antigens such as CMV, associated immune responses, ART or a combination of all these factors? What do these findings mean in terms of immunological health in early childhood? Long-term outcomes for these infants need to be described through clinical and developmental follow-up, particularly for HEU infants with cCMV with or without symptoms at birth. Outcomes need to be better and more comprehensively defined, including mortality, risk for early childhood infection, growth and neurodevelopment delays. A composite picture of such outcomes, combined with molecular and immunological investigations, will inform prevention and management approaches relevant for antenatal, neonatal and paediatric care.

## 3. A synthesis of dots

We have described how HIV and CMV independently disrupt normal physiological processes of pregnancy, with their effects magnified in HIV–CMV coinfection. Key events leading to disruption of the maternal–fetal interface, transmission of infection and/or inflammation and adverse infant outcomes are probably influenced by the immunological function of the placenta and the balance between inflationary and non-inflationary CD8+ T-cell responses during pregnancy.

CD8+ T cells are critical mediators of protective immunity. However, both HIV and CMV are known to cause persistent immune activation, expanding CD8+ T cells with an exhausted phenotype. This dysfunction is often evident despite ART-mediated HIV suppression and may relate to duration of HIV infection prior to treatment. We have seen that pre-conceptual immunity to CMV does reduce CMV transmission but does not completely prevent it. This may be dependent on the state of differentiation of CMV-specific adaptive responses and activation of tissue-resident memory CD8+ and CD4+ T cells. Understanding the determinants of late differentiation and dynamics between CMV latent reservoirs, viral reactivation and CMV-driven T-cell exhaustion may be key to detecting non-primary transmission. This could enable timely interventions to prevent fetal disease through the use of vaccines or targeted antiviral therapy during early pregnancy. Specifically, knowing whether a more exhausted phenotype correlates with decreased T-cell function and thereby increased likelihood of CMV transmission may help identify a subgroup of women at higher risk.

CMV infection at the maternal–fetal interface can impair blood flow, disrupt spiral artery remodelling, induce local hypoxia and cause damage to placental cells. This is probably driven by heightened immune responses in early gestation, characterized by infiltration of inflammatory cells and the release of pro-inflammatory mediators that subsequently impair materno-fetal vasculature. However, the magnitude of CMV-driven T-cell activation is also influenced by maternal HLA allele expression, owing to the critical role that these alleles have in recognition of and immune response to CMV. Functional analysis has revealed that HIV uniquely associates with upregulation of reactive oxygen species that cause cellular damage, and CMV uniquely associates with disruption of the WNT/β-Catenin signalling pathway [87]. This poses the intriguing hypothesis that the neurological deficits observed from first trimester CMV infection may be driven by poor placental angiogenesis and pathology.

Within the placenta, themes with translational implications that warrant further investigation include:

(1) How CMV–HIV coinfection affects placental micro-anatomy and immune networks;
(2) The role of maternal–infant HLA allele sharing in either controlling or promoting CMV transmission;
(3) Whether microchimeric transmission of CD8+ T cells across the placenta has a protective or pathogenic role, i.e. do these cells contribute to infection control or instead mediate inflammatory damage?

Perhaps philosophically, the offshoot of an attempted protective antenatal CD8+ T cells response in some individuals is that these cells cross into the placenta and inadvertently cause placental pathology.

In summary, the key factors underpinning CMV transmission probably involve the interplay between maternal CMV viraemia, CD8+ T-cell function and placental immune activation. It is crucial that aligned mother–infant studies continue to explore these dynamics, capturing a full spectrum of infant outcomes including survival, immune development, growth and hearing outcomes in early childhood. These efforts will enhance our understanding of non-primary CMV transmission, which is a neglected area accounting for the majority of cCMV cases in low- and middle-income countries. Identifying biomarkers for non-primary CMV could enable early antiviral therapy to prevent vertical transmission. Such knowledge would also inform vaccine development and novel therapeutics aimed at curbing inflammation-driven transmission. Ultimately, better CMV management during pregnancy will reduce cCMV incidence, enable earlier diagnosis and intervention, and decrease rates of childhood hearing loss and disability.

## Data Availability

All data referred to in this article have been published elsewhere and are cited accordingly.

## References

[1] Ssentongo P *et al*. 2021 Congenital cytomegalovirus infection burden and epidemiologic risk factors in countries with universal screening. JAMA Netw. Open **4**, e2120736. (10.1001/jamanetworkopen.2021.20736) 34424308 PMC8383138

[2] Jones CE *et al*. 2023 Managing challenges in congenital CMV: current thinking. Arch. Dis. Child. **108**, 601–607. (10.1136/archdischild-2022-323809) 36442957

[3] Zuhair M, Smit GSA, Wallis G, Jabbar F, Smith C, Devleesschauwer B, Griffiths P. 2019 Estimation of the worldwide seroprevalence of cytomegalovirus: a systematic review and meta‐analysis. Rev. Med. Virol **29**, e2034. (10.1002/rmv.2034) 30706584

[4] Flanders WD, Lally C, Dilley A, Diaz‐Decaro J. 2024 Estimated cytomegalovirus seroprevalence in the general population of the United States and Canada. J. Med. Virol. **96**, e29525. (10.1002/jmv.29525) 38529529

[5] Zhao M, Zhuo C, Li Q, Liu L. 2020 Cytomegalovirus (CMV) infection in HIV/AIDS patients and diagnostic values of CMV-DNA detection across different sample types. Ann. Cardiothorac. Surg **9**, 2710–2715. (10.21037/apm-20-1352) 32819135

[6] Ballegaard V, Brændstrup P, Pedersen KK, Kirkby N, Stryhn A, Ryder LP, Gerstoft J, Nielsen SD. 2018 Cytomegalovirus-specific T-cells are associated with immune senescence, but not with systemic inflammation, in people living with HIV. Sci. Rep. **8**, 1–13. (10.1038/s41598-018-21347-4) 29491459 PMC5830877

[7] Slyker JA *et al*. 2009 The detection of cytomegalovirus DNA in maternal plasma is associated with mortality in HIV-1-infected women and their infants. AIDS **23**, 117–124. (10.1097/qad.0b013e32831c8abd) 19050393 PMC2739581

[8] Astawesegn FH, Stulz V, Conroy E, Mannan H. 2022 Trends and effects of antiretroviral therapy coverage during pregnancy on mother-to-child transmission of HIV in Sub-Saharan Africa. Evidence from panel data analysis. BMC Infect. Dis. **22**, 134. (10.1186/s12879-022-07119-6) 35135474 PMC8822759

[9] Enders G, Daiminger A, Bäder U, Exler S, Enders M. 2011 Intrauterine transmission and clinical outcome of 248 pregnancies with primary cytomegalovirus infection in relation to gestational age. J. Clin. Virol. **52**, 244–246. (10.1016/j.jcv.2011.07.005) 21820954

[10] Faure-Bardon V *et al*. 2019 Sequelae of congenital cytomegalovirus following maternal primary infections are limited to those acquired in the first trimester of pregnancy. Clin. Infect. Dis. **69**, 1526–1532. (10.1093/cid/ciy1128) 30596974

[11] Chatzakis C, Ville Y, Makrydimas G, Dinas K, Zavlanos A, Sotiriadis A. 2020 Timing of primary maternal cytomegalovirus infection and rates of vertical transmission and fetal consequences. Am. J. Obstet. Gynecol. **223**, 870–883. (10.1016/j.ajog.2020.05.038) 32460972

[12] Forte E, Zhang Z, Thorp EB, Hummel M. 2020 Cytomegalovirus latency and reactivation: an intricate interplay with the host immune response. Front. Cell. Infect. Microbiol. **10**, 130. (10.3389/fcimb.2020.00130) 32296651 PMC7136410

[13] Umashankar M *et al*. 2011 A novel human cytomegalovirus locus modulates cell type-specific outcomes of infection. PLoS Pathog. **7**, e1002444. (10.1371/journal.ppat.1002444) 22241980 PMC3248471

[14] Poole E, Lau J, Groves I, Roche K, Murphy E, Carlan da Silva M, Reeves M, Sinclair J. 2023 The human cytomegalovirus latency-associated gene product latency unique natural antigen regulates latent gene expression. Viruses **15**, 1875. (10.3390/v15091875) 37766281 PMC10536386

[15] Yamamoto AY, Mussi-Pinhata MM, Boppana SB, Novak Z, Wagatsuma VM, Oliveira P de F, Duarte G, Britt WJ. 2010 Human cytomegalovirus reinfection is associated with intrauterine transmission in a highly cytomegalovirus-immune maternal population. Am. J. Obstet. Gynecol **297**, e1–8. (10.1016/j.ajog.2009.11.018) PMC8351475 20060091

[16] Simonazzi G *et al*. 2018 Perinatal outcomes of non-primary maternal cytomegalovirus infection: a 15-year experience. Fetal Diagn. Ther. **43**, 138–142. (10.1159/000477168) 28697499

[17] Boppana SB, Rivera LB, Fowler KB, Mach M, Britt WJ. 2001 Intrauterine transmission of cytomegalovirus to infants of women with preconceptional immunity. N. Engl. J. Med. **344**, 1366–1371. (10.1056/nejm200105033441804) 11333993

[18] Huang Y *et al*. 2022 Pre-existing maternal IgG antibodies as a protective factor against congenital cytomegalovirus infection: a mother-child prospective cohort study. eBioMedicine **77**, 103885. (10.1016/j.ebiom.2022.103885) 35183868 PMC8861648

[19] Alfi O *et al*. 2024 Decidual-tissue-resident memory T cells protect against nonprimary human cytomegalovirus infection at the maternal-fetal interface. Cell Rep. **43**, 113698. (10.1016/j.celrep.2024.113698) 38265934

[20] Leruez-Ville M, Magny JF, Couderc S, Pichon C, Parodi M, Bussières L, Guilleminot T, Ghout I, Ville Y. 2017 Risk factors for congenital cytomegalovirus infection following primary and nonprimary maternal infection. Clin. Infect. Dis. **65**, 398–404. (10.1093/cid/cix337) 28419213

[21] Sun L *et al*. 2022 Cytomegalovirus cell tropism and clinicopathological characteristics in gastrointestinal tract of patients with HIV/AIDS. Diagn. Pathol. **17**, 1–8. (10.1186/s13000-022-01193-9) 35027044 PMC8759214

[22] Cimato G, Zhou X, Brune W, Frascaroli G. 2024 Human cytomegalovirus glycoprotein variants governing viral tropism and syncytium formation in epithelial cells and macrophages. J. Virol. **98**, e0029324. (10.1128/jvi.00293-24) 38837351 PMC11265420

[23] Gordon CL *et al*. 2017 Tissue reservoirs of antiviral T cell immunity in persistent human CMV infection. J. Exp. Med. **214**, 651–667. (10.1084/jem.20160758) 28130404 PMC5339671

[24] Sylwester AW *et al*. 2005 Broadly targeted human cytomegalovirus-specific CD4^+^ and CD8^+^ T cells dominate the memory compartments of exposed subjects. J. Exp. Med. **202**, 673–685. (10.1084/jem.20050882) 16147978 PMC2212883

[25] Dhanwani R *et al*. 2021 Profiling human cytomegalovirus-specific T cell responses reveals novel immunogenic open reading frames. J. Virol. **95**, e0094021. (10.1128/JVI.00940-21) 34379494 PMC8513490

[26] van den Berg SPH *et al*. 2021 Quantification of T-cell dynamics during latent cytomegalovirus infection in humans. PLoS Pathog. **17**, 1–27. (10.1371/journal.ppat.1010152) PMC8717968 34914799

[27] Martin-Ruiz C *et al*. 2020 CMV-independent increase in CD27-CD28^+^ CD8^+^ EMRA T cells is inversely related to mortality in octogenarians. NPJ Aging Mech. Dis. **6**, 1–6. (10.1038/s41514-019-0041-y) 31993214 PMC6972903

[28] Redeker A *et al*. 2018 The contribution of cytomegalovirus infection to immune senescence is set by the infectious dose. Front. Immunol. **8**, 1953. (10.3389/fimmu.2017.01953) 29367854 PMC5768196

[29] Lindau P, Mukherjee R, Gutschow MV, Vignali M, Warren EH, Riddell SR, Makar KW, Turtle CJ, Robins HS. 2019 Cytomegalovirus exposure in the elderly does not reduce CD8 T cell repertoire diversity. J. Immunol. **202**, 476–483. (10.4049/jimmunol.1800217) 30541882 PMC6321841

[30] Kallemeijn MJ, Boots AMH, van der Klift MY, Brouwer E, Abdulahad WH, Verhaar JAN, van Dongen JJM, Langerak AW. 2017 Ageing and latent CMV infection impact on maturation, differentiation and exhaustion profiles of T-cell receptor gammadelta T-cells. Sci. Rep. **7**, 1–14. (10.1038/s41598-017-05849-1) 28710491 PMC5511140

[31] O’Hara GA, Welten SPM, Klenerman P, Arens R. 2012 Memory T cell inflation: understanding cause and effect. Trends Immunol. **33**, 84–90. (10.1016/j.it.2011.11.005) 22222196

[32] Abassi L, Cicin-Sain L. 2020 The avid competitors of memory inflation. Curr. Opin. Virol. **44**, 162–168. (10.1016/j.coviro.2020.08.007) 33039898

[33] Zangger N, Oxenius A. 2022 T cell immunity to cytomegalovirus infection. Curr. Opin. Immunol. **77**, 102185. (10.1016/j.coi.2022.102185) 35576865

[34] Huygens A *et al*. 2015 Functional exhaustion limits CD4^+^ and CD8^+^ T-cell responses to congenital cytomegalovirus infection. J. Infect. Dis. **212**, 484–494. (10.1093/infdis/jiv071) 25657256

[35] Kim HG *et al*. 2025 Significance of placental pathology and CMV PCR in assessing clinical outcomes of congenital CMV infection. Placenta **167**, 22–27. (10.1016/j.placenta.2025.04.020) 40311175

[36] Rollman TB, Berkebile ZW, Okae H, Bardwell VJ, Gearhart MD, Bierle CJ. 2024 Human trophoblast stem cells restrict human cytomegalovirus replication. J. Virol. **98**, e01935-23. (10.1128/jvi.01935-23) 38451085 PMC11019952

[37] Girsch JH *et al*. 2022 Host-viral interactions at the maternal-fetal interface. What we know and what we need to know. Front. Virol. **2**, 2022.833106. (10.3389/fviro.2022.833106) PMC9894500 36742289

[38] Liu T *et al*. 2015 Role of human cytomegalovirus in the proliferation and invasion of extravillous cytotrophoblasts isolated from early placentae. Int. J. Clin. Exp. Med. **8**, 17248–17260. https://pubmed.ncbi.nlm.nih.gov/26770317/26770317 PMC4694217

[39] Mor G, Cardenas I, Abrahams V, Guller S. 2011 Inflammation and pregnancy: the role of the immune system at the implantation site. Ann. N. Y. Acad. Sci. **1221**, 80–87. (10.1111/j.1749-6632.2010.05938.x) 21401634 PMC3078586

[40] Jun Y, Kim E, Jin M, Sung HC, Han H, Geraghty DE, Ahn K. 2000 Human cytomegalovirus gene products US3 and US6 down-regulate trophoblast class I MHC molecules. J. Immunol. **164**, 805–811. (10.4049/jimmunol.164.2.805) 10623826

[41] Crespo ÂC, Strominger JL, Tilburgs T. 2016 Expression of KIR2DS1 by decidual natural killer cells increases their ability to control placental HCMV infection. Proc. Natl Acad. Sci. USA **113**, 15072–15077. (10.1073/pnas.1617927114) 27956621 PMC5206558

[42] Mimura N *et al*. 2022 Suppression of human trophoblast syncytialization by human cytomegalovirus infection. Placenta **117**, 200–208. (10.1016/j.placenta.2021.12.011) 34933151

[43] Chou D, Ma Y, Zhang J, McGrath C, Parry S. 2006 Cytomegalovirus infection of trophoblast cells elicits an inflammatory response: a possible mechanism of placental dysfunction. Am. J. Obstet. Gynecol. **194**, 535–541. (10.1016/j.ajog.2005.07.073) 16458658

[44] Bates M, Brantsaeter AB. 2016 Human cytomegalovirus (CMV) in Africa: a neglected but important pathogen. J. Virus Erad. **2**, 136–142. (10.1016/s2055-6640(20)30456-8) 27482452 PMC4967964

[45] Payne H, Barnabas S. 2024 Congenital cytomegalovirus in Sub-Saharan Africa—a narrative review with practice recommendations. Front. Public Health **12**, 1359663. (10.3389/fpubh.2024.1359663) 38813410 PMC11134569

[46] Johnson EL, Boggavarapu S, Johnson ES, Lal AA, Agrawal P, Bhaumik SK, Murali-Krishna K, Chakraborty R. 2018 Human cytomegalovirus enhances placental susceptibility and replication of human immunodeficiency virus type 1 (HIV-1), which may facilitate in utero HIV-1 transmission. J. Infect. Dis. **218**, 1464–1473. (10.1093/infdis/jiy327) 29860306 PMC6927849

[47] Otieno NA *et al*. 2019 The impact of maternal HIV and malaria infection on the prevalence of congenital cytomegalovirus infection in Western Kenya. J. Clin. Virol. **120**, 33–37. (10.1016/j.jcv.2019.09.007) 31546088 PMC6815230

[48] Duri K *et al*. 2021 Role of antenatal plasma cytomegalovirus DNA levels on pregnancy outcome and HIV-1 vertical transmission among mothers in the University of Zimbabwe birth cohort study (UZBCS). Virol. J. **18**, 30. (10.1186/s12985-021-01494-3) 33514390 PMC7846993

[49] Olmos-Ortiz A, Flores-Espinosa P, Mancilla-Herrera I, Vega-Sánchez R, Díaz L, Zaga-Clavellina V. 2019 Innate immune cells and Toll-like receptor–dependent responses at the maternal–fetal interface. Int. J. Mol. Sci. **20**, 3654. (10.3390/ijms20153654) 31357391 PMC6695670

[50] Pfeifer C, Bunders MJ. 2016 Maternal HIV infection alters the immune balance in the mother and fetus; implications for pregnancy outcome and infant health. Curr. Opin. HIV AIDS **11**, 138–145. (10.1097/coh.0000000000000239) 26679415

[51] Pereira NZ *et al*. 2020 Increased expression on innate immune factors in placentas from HIV-infected mothers concurs with dampened systemic immune activation. Front. Immunol. **11**, 1822. (10.3389/fimmu.2020.01822) 32983090 PMC7477039

[52] Weckman AM, Ngai M, Wright J, McDonald CR, Kain KC. 2019 The impact of infection in pregnancy on placental vascular development and adverse birth outcomes. Front. Microbiol. **10**, 1924. (10.3389/fmicb.2019.01924) 31507551 PMC6713994

[53] Chan G, Guilbert LJ. 2006 Ultraviolet-inactivated human cytomegalovirus induces placental syncytiotrophoblast apoptosis in a Toll-like receptor-2 and tumour necrosis factor-alpha dependent manner. J. Pathol. **210**, 111–120. (10.1002/path.2025) 16826536

[54] Kavathas PB, Boeras CM, Mulla MJ, Abrahams VM. 2013 Nod1, but not the ASC inflammasome, contributes to induction of IL-1β secretion in human trophoblasts after sensing of Chlamydia trachomatis. Mucosal Immunol. **6**, 235–243. (10.1038/mi.2012.63) 22763410 PMC3465624

[55] Pontillo A, Girardelli M, Agostinis C, Masat E, Bulla R, Crovella S. 2013 Bacterial LPS differently modulates inflammasome gene expression and IL-1β secretion in trophoblast cells, decidual stromal cells, and decidual endothelial cells. Reprod. Sci. **20**, 563–566. (10.1177/1933719112459240) 23184659

[56] Stødle GS *et al*. 2018 Placental inflammation in pre-eclampsia by Nod-like receptor protein (NLRP)3 inflammasome activation in trophoblasts. Clin. Exp. Immunol. **193**, 84–94. (10.1111/cei.13130) 29683202 PMC6038006

[57] Patni S, Wynen LP, Seager AL, Morgan G, White JO, Thornton CA. 2009 Expression and activity of Toll-like receptors 1–9 in the human term placenta and changes associated with labor at term. Biol. Reprod. **80**, 243–248. (10.1095/biolreprod.108.069252) 18815357

[58] Tangerås LH *et al*. 2014 Functional Toll-like receptors in primary first-trimester trophoblasts. J. Reprod. Immunol. **106**, 89–99. (10.1016/j.jri.2014.04.004) 24933117

[59] Koga K, Aldo PB, Mor G. 2009 Toll‐like receptors and pregnancy: trophoblast as modulators of the immune response. J. Obstet. Gynaecol. Res. **35**, 191–202. (10.1111/j.1447-0756.2008.00963.x) 19335792

[60] Renaud SJ, Cotechini T, Quirt JS, Macdonald-Goodfellow SK, Othman M, Graham CH. 2011 Spontaneous pregnancy loss mediated by abnormal maternal inflammation in rats is linked to deficient uteroplacental perfusion. J. Immunol. **186**, 1799–1808. (10.4049/jimmunol.1002679) 21187445

[61] Taylor SA, Sharma S, Remmel CAL, Holder B, Jones CE, Marchant A, Ackerman ME. 2022 HIV-associated alterations of the biophysical features of maternal antibodies correlate with their reduced transfer across the placenta. J. Infect. Dis. **226**, 1441–1450. (10.1093/infdis/jiac222) 35668706 PMC9574667

[62] Hemmings DG, Kilani R, Nykiforuk C, Preiksaitis J, Guilbert LJ. 1998 Permissive cytomegalovirus infection of primary villous term and first trimester trophoblasts. J. Virol. **72**, 4970–4979. (10.1128/jvi.72.6.4970-4979.1998) 9573266 PMC110059

[63] Pereira L *et al*. 2014 Intrauterine growth restriction caused by underlying congenital cytomegalovirus infection. J. Infect. Dis. **209**, 1573–1584. (10.1093/infdis/jiu019) 24403553 PMC3997585

[64] Azamor T *et al*. 2024 Transplacental SARS-CoV-2 protein ORF8 binds to complement C1q to trigger fetal inflammation. EMBO J. **43**, 5494–5529. (10.1038/s44318-024-00260-9) 39390219 PMC11574245

[65] van der Zwan A, Bi K, Norwitz ER, Crespo ÂC, Claas FHJ, Strominger JL, Tilburgs T. 2018 Mixed signature of activation and dysfunction allows human decidual CD8^+^ T cells to provide both tolerance and immunity. Proc. Natl Acad. Sci. USA **115**, 385–390. (10.1073/pnas.1713957115) 29259116 PMC5777048

[66] Kim CJ, Romero R, Chaemsaithong P, Kim JS. 2015 Chronic inflammation of the placenta: definition, classification, pathogenesis, and clinical significance. Am. J. Obstet. Gynecol. **213**, S53–S69. (10.1016/j.ajog.2015.08.041) 26428503 PMC4782598

[67] Ikumi NM *et al*. 2021 T-Cell homeostatic imbalance in placentas from women with human immunodeficiency virus in the absence of vertical transmission. J. Infect. Dis. **224**, S670–S682. (10.1093/infdis/jiab192) 33880544 PMC8883807

[68] López M, Figueras F, Coll O, Goncé A, Hernández S, Loncá M, Vila J, Gratacós E, Palacio M. 2016 Inflammatory markers related to microbial translocation among HIV-infected pregnant women: a risk factor of preterm delivery. J. Infect. Dis. **213**, 343–350. (10.1093/infdis/jiv416) 26265778

[69] Truong HHM, Sim MS, Dillon M, Uittenbogaart CH, Dickover R, Plaeger SF, Bryson YJ. 2010 Correlation of immune activation during late pregnancy and early postpartum with increases in plasma HIV RNA, CD4/CD8 T cells, and serum activation markers. Clin. Vaccine Immunol. **17**, 2024–2028. (10.1128/cvi.00088-10) 20980480 PMC3008186

[70] Altfeld M, Bunders MJ. 2016 Impact of HIV-1 infection on the feto-maternal crosstalk and consequences for pregnancy outcome and infant health. Semin. Immunopathol. **38**, 727–738. (10.1007/s00281-016-0578-9) 27392971

[71] Wedi COO, Kirtley S, Hopewell S, Corrigan R, Kennedy SH, Hemelaar J. 2016 Perinatal outcomes associated with maternal HIV infection: a systematic review and meta-analysis. Lancet HIV **3**, e33–e48. (10.1016/s2352-3018(15)00207-6) 26762992

[72] Papp E *et al*. 2015 HIV protease inhibitor use during pregnancy is associated with decreased progesterone levels, suggesting a potential mechanism contributing to fetal growth restriction. J. Infect. Dis. **211**, 10–18. (10.1093/infdis/jiu393) 25030058 PMC4264589

[73] Papp E *et al*. 2016 Low prolactin and high 20-α-hydroxysteroid dehydrogenase levels contribute to lower progesterone levels in HIV-infected pregnant women exposed to protease inhibitor-based combination antiretroviral therapy. J. Infect. Dis. **213**, 1532–1540. (10.1093/infdis/jiw004) 26740274 PMC4837912

[74] Kala S, Dunk C, Acosta S, Serghides L. 2020 Periconceptional exposure to lopinavir, but not darunavir, impairs decidualization: a potential mechanism leading to poor birth outcomes in HIV-positive pregnancies. Hum. Reprod. **35**, 1781–1796. (10.1093/humrep/deaa151) 32712670 PMC7398624

[75] Savid-Frontera C, Viano ME, Baez NS, Lidon NL, Fontaine Q, Young HA, Vimeux L, Donnadieu E, Rodriguez-Galan MC. 2022 Exploring the immunomodulatory role of virtual memory CD8^+^ T cells: role of IFN gamma in tumor growth control. Front. Immunol. **13**, 1–18. (10.3389/fimmu.2022.971001) PMC9623162 36330506

[76] Lee JY, Hamilton SE, Akue AD, Hogquist KA, Jameson SC. 2013 Virtual memory CD8 T cells display unique functional properties. Proc. Natl Acad. Sci. USA **110**, 13498–13503. (10.1073/pnas.1307572110) 23898211 PMC3746847

[77] Rolot M *et al*. 2018 Helminth-induced IL-4 expands bystander memory CD8^+^ T cells for early control of viral infection. Nat. Commun. **9**, 4516. (10.1038/s41467-018-06978-5) 30375396 PMC6207712

[78] Clerici M *et al*. 2000 T-lymphocyte maturation abnormalities in uninfected newborns and children with vertical exposure to HIV. Blood **96**, 3866–3871. (10.1182/blood.v96.12.3866.h8003866_3866_3871) 11090071

[79] Bunders MJ, van Hamme JL, Jansen MH, Boer K, Kootstra NA, Kuijpers TW. 2014 Fetal exposure to HIV-1 alters chemokine receptor expression by CD4^+^T cells and increases susceptibility to HIV-1. Sci. Rep. **4**, 6690. (10.1038/srep06690) 25341640 PMC4208038

[80] Tilburgs T, Strominger JL. 2013 CD8+ effector T cells at the fetal–maternal interface, balancing fetal tolerance and antiviral immunity. Am. J. Reprod. Immunol. **69**, 395–407. (10.1111/aji.12094) 23432707 PMC3711858

[81] Mahajan S *et al*. 2023 Antigen-specific decidual CD8^+^ T cells include distinct effector memory and tissue-resident memory cells. JCI Insight **8**, e171806. (10.1172/jci.insight.171806) 37681414 PMC10544202

[82] van der Zwan A, Bi K, Norwitz ER, Crespo ÂC, Claas FHJ, Strominger JL, Tilburgs T. 2018 T cells to provide both tolerance and immunity. Proc. Natl Acad. Sci. USA **115**, 385–390. (10.1073/pnas.1713957115) 29259116 PMC5777048

[83] van Egmond A, van der Keur C, Swings GMJS, Scherjon SA, Claas FHJ. 2016 The possible role of virus-specific CD8^+^ memory T cells in decidual tissue. J. Reprod. Immunol. **113**, 1–8. (10.1016/j.jri.2015.09.073) 26496155

[84] Johnson EL, Chakraborty R. 2012 Placental Hofbauer cells limit HIV-1 replication and potentially offset mother to child transmission (MTCT) by induction of immunoregulatory cytokines. Retrovirology **9**, 101. (10.1186/1742-4690-9-101) 23217137 PMC3524025

[85] Pereira L, Maidji E, McDonagh S, Tabata T. 2005 Insights into viral transmission at the uterine–placental interface. Trends Microbiol. **13**, 164–174. (10.1016/j.tim.2005.02.009) 15817386

[86] Swieboda D *et al*. 2020 Baby’s first macrophage: temporal regulation of Hofbauer cell phenotype influences ligand-mediated innate immune responses across gestation. J. Immunol. **204**, 2380–2391. (10.4049/jimmunol.1901185) 32213562 PMC7870092

[87] Schuch V, Hossack D, Hailstorks T, Chakraborty R, Johnson EL. 2024 Distinct immune responses to HIV and CMV in Hofbauer cells across gestation highlight evolving placental immune dynamics. BioRxiv 2024.11.21.624730. (10.1101/2024.11.21.624730) PMC13286909 42344922

[88] Angelova M, Zwezdaryk K, Ferris M, Shan B, Morris CA, Sullivan DE. 2012 Human cytomegalovirus infection dysregulates the canonical wnt/β-catenin signaling pathway. PLoS Pathog. **8**, e1002959. (10.1371/journal.ppat.1002959) 23071438 PMC3469659

[89] Tabata T, Petitt M, Zydek M, Fang-Hoover J, Larocque N, Tsuge M, Gormley M, Kauvar LM, Pereira L. 2015 Human cytomegalovirus infection interferes with the maintenance and differentiation of trophoblast progenitor cells of the human placenta. J. Virol. **89**, 5134–5147. (10.1128/jvi.03674-14) 25741001 PMC4403461

[90] Chen JC. 2021 Immunological consequences of in utero exposure to foreign antigens. Front. Immunol. **12**, 638435. (10.3389/fimmu.2021.638435) 33936052 PMC8082100

[91] Johnson EL, Swieboda D, Olivier A, Enninga EAL, Chakraborty R. 2021 Robust innate immune responses at the placenta during early gestation may limit in utero HIV transmission. PLoS Pathog. **17**, e1009860. (10.1371/journal.ppat.1009860) 34432853 PMC8437274

[92] Miller D *et al*. 2023 Immunosequencing and profiling of T cells at the maternal–fetal interface of women with preterm labor and chronic chorioamnionitis. J. Immunol. **211**, 1082–1098. (10.4049/jimmunol.2300201) 37647360 PMC10528178

[93] Tsuda S, Nakashima A, Morita K, Shima T, Yoneda S, Kishi H, Saito S. 2021 The role of decidual regulatory T cells in the induction and maintenance of fetal antigen-specific tolerance: imbalance between regulatory and cytotoxic T cells in pregnancy complications. Hum. Immunol. **82**, 346–352. (10.1016/j.humimm.2021.01.019) 33642099

[94] Prendergast AJ, Klenerman P, Goulder PJR. 2012 The impact of differential antiviral immunity in children and adults. Nat. Rev. Immunol. **12**, 636–648. (10.1038/nri3277) 22918466

[95] Wang J, Han T, Zhu X. 2024 Role of maternal–fetal immune tolerance in the establishment and maintenance of pregnancy. Chin. Med. J. **137**, 1399–1406. (10.1097/CM9.0000000000003114) 38724467 PMC11188918

[96] Weisblum Y, Panet A, Haimov-Kochman R, Wolf DG. 2014 Models of vertical cytomegalovirus (CMV) transmission and pathogenesis. Semin. Immunopathol. **36**, 615–625. (10.1007/s00281-014-0449-1) 25291972

[97] Ouellette CP *et al*. 2020 Blood genome expression profiles in infants with congenital cytomegalovirus infection. Nat. Commun. **11**, 3548. (10.1038/s41467-020-17178-5) 32669541 PMC7363904

[98] Capretti MG, Marsico C, Chiereghin A, Gabrielli L, Aceti A, Lazzarotto T. 2021 Immune monitoring using QuantiFERON®-CMV assay in congenital cytomegalovirus infection: correlation with clinical presentation and CMV DNA load. Clin. Infect. Dis. **73**, 367–373. (10.1093/cid/ciaa704) 32504086

[99] Medoro AK *et al*. 2024 T cell responses and clinical symptoms among infants with congenital cytomegalovirus infection. JCI Insight **9**, e171029. (10.1172/jci.insight.171029) 39315550 PMC11457853

[100] Lwanga C *et al*. 2024 Impact of HIV exposure without infection on hospital course and mortality among young children in sub-Saharan Africa: a multi-site cohort study. BMC Med. **22**, 573. (10.1186/s12916-024-03790-5) 39627711 PMC11613948

[101] Brochon J *et al*. 2025 Increased risk of hospitalization among children who were HIV-exposed and uninfected compared to population controls. AIDS **39**, 40–48. (10.1097/QAD.0000000000004025) 39329518

[102] Dzanibe S, Wilk AJ, Canny S. 2024 Premature skewing of T cell receptor clonality and delayed memory expansion in HIV-exposed infants. Nat. Commun. **15**, 4080. (10.1038/s41467-024-47955-5) 38744812 PMC11093981

